# Predictors of kidney function recovery among incident ESRD patients

**DOI:** 10.1186/s12882-021-02345-7

**Published:** 2021-04-21

**Authors:** Maria Santos, Huiying Yin, Diane Steffick, Rajiv Saran, Michael Heung

**Affiliations:** 1 University of Michigan Medical School, 1500 E. Medical Center Drive, SPC 5364, Ann Arbor, MI 48109-5364 USA; 2 University of Michigan Kidney Epidemiology and Cost Center, Ann Arbor, USA; 3 Department of Medicine, Division of Nephrology, University of Michigan, Ann Arbor, USA

**Keywords:** End-stage renal disease, Acute kidney injury, Kidney recovery, Risk factors

## Abstract

**Background:**

ESRD is considered an irreversible loss of renal function, yet some patients will recover kidney function sufficiently to come off dialysis. Potentially modifiable predictors of kidney recovery, such as dialysis prescription, have not been fully examined.

**Methods:**

Retrospective cohort study using United States Renal Data System (USRDS) data to identify incident hemodialysis (HD) patients between 2012 and 2016, the first 4 years for which dialysis treatment data is available. The primary outcome was kidney recovery within 1 year of ESRD and HD initiation, defined by a specific recovery code and survival off dialysis for at least 30 days. Patient and treatment characteristics were compared between those that recovered versus those that remained dialysis-dependent. A time-dependent survival model was used to identify independent predictors of kidney recovery.

**Results:**

During the study period, there were 372,387 incident HD patients with available data, among whom 16,930 (4.5%) recovered to dialysis-independence. Compared to non-recovery, a higher proportion of patients with kidney recovery were of white race, and non-Hispanic ethnicity. Both groups had a similar age distribution. Patients with an acute kidney injury diagnosis as primary cause of ESRD were most likely to recover, but the most common ESRD diagnosis among recovering patients was type 2 diabetes (29.8% of recovery cases). Higher eGFR and lower albumin at ESRD initiation were associated with increased likelihood of recovery. When examining HD ultrafiltration rate (UFR), each quintile above the first quintile was associated with a progressively lower likelihood of recovery (HR 0.45, 95% CI 0.43–0.48 for highest versus lowest quintile, *p* < 0.001).

**Conclusions:**

We identified non-modifiable and potentially modifiable factors associated with kidney recovery which may assist clinicians in counseling and monitoring incident ESRD patients with a greater chance to gain dialysis-independence. Clinical trials are warranted to examine the impact of dialysis prescription on subsequent kidney function recovery.

## Background

By definition, a diagnosis of end-stage renal disease (ESRD) indicates that patients have irreversibly lost kidney function to the point of requiring dialysis or transplantation. Yet a proportion of such patients will in fact recover kidney function sufficient to come off dialysis, at least for a period of time – as high as 5–6% of incident ESRD patients in recent years [1, 2]. Dialysis-independence is a critically important and highly patient-centered outcome for dialysis patients, but there are currently no tools to help clinicians more accurately identify which patients are most likely to recover.

Because ESRD designation is a clinical determination, it remains prone to some inherent imprecision. Identification of patient factors associated with recovery can help guide both patient and provider expectations and assist in monitoring for recovery. This can be important because kidney function recovery in the dialysis center may not be recognized if not actively searched for. In many dialysis centers, it is not routine to assess residual kidney function and therefore any improvement in kidney function may be missed. Under-recognition of recovery can result in patients remaining on dialysis longer than necessary.

Perhaps even more importantly, there is a need to identify clinical practices – including dialysis prescription – that may influence (either positively or negatively) the chances of kidney recovery. The potential negative impacts of hemodialysis therapy on cardiac and neurologic function have been well-described [3, 4], and this may translate to exacerbating kidney injury when kidney recovery is still possible. Dialysis treatment factors have not been examined in the context of ESRD recovery, in part because of limited availability of such data. However, in 2012, mandatory reporting of some clinical treatment data from U.S. dialysis centers began through the Consolidated Renal Operations in a Web Enabled Network (CROWNWeb) application, providing an opportunity to examine dialysis prescription factors with outcomes on a national level.

The goal of this study was to identify ESRD patient characteristics and hemodialysis prescription factors associated with subsequent kidney function recovery, with the hope of informing current practices as well as laying the foundation for further research into optimizing treatment approaches in this patient population.

## Methods

### Data sources and patient population

The United States Renal Data System (USRDS) collects, analyzes, and distributes information about chronic kidney disease (CKD) and ESRD in the United States. The USRDS data sources include Centers for Medicare & Medicaid Services (CMS), the United Network for Organ Sharing (UNOS), and selected clinical and administrative data reported monthly by all Medicare-certified dialysis facilities in the U.S. (CROWNWeb). Data from these sources are merged together to create a treatment history file of ESRD treatment for each patient [5, 6]. This study was approved by the University of Michigan institutional review board with a waiver of consent due to the retrospective nature and use of de-identified datasets (HUM00086162).

We conducted a retrospective cohort study of incident ESRD patients on dialysis in the U.S. We identified patients with kidney recovery by ESRD initiation year from 1996 through 2016 in order to describe trends. We then focused the cohort analysis on patients starting maintenance hemodialysis (HD) between May 1st, 2012 and May 31st, 2016, representing the initial period for which CROWNWeb treatment data became available. Patient treatments were followed through May 31st, 2017, allowing at least 1-year of follow-up on all patients. Patient demographic information, including age, sex, race, and ethnicity were obtained from USRDS 2017 Standard Analysis Files (SAF). Additional clinical information, such as pre-ESRD laboratory data, insurance coverage, cause of ESRD and pre-ESRD care, were obtained from the ESRD medical evidence Form (CMS Form 2728).

Hemodialysis treatment data was obtained from CROWNWeb. Data are reported to CROWNWeb on a once-monthly data and represent the findings from a single treatment, as opposed to the average of all monthly treatments. Dialysis ultrafiltration rate (UFR) was calculated as (pre-HD weight – post-HD weight)/(post-HD weight)/(treatment time) for each month since ESRD initiation. The mean UFR of the first 12 months was grouped into quintiles and used in the descriptive analysis. The monthly UFR was also grouped into separate quintiles and used in the time-dependent model.

Our primary outcome was recovery to dialysis independence within 1 year of ESRD initiation. ESRD recovery events were identified by the recovery of kidney function code in the USRDS patient event file, and patients had to survive at least 30 days without receiving additional dialysis or undergoing kidney transplantation. This parameter was an effort to exclude patients that were withdrawn from dialysis. Of note, there is a separate specific code to denote withdrawal from dialysis for palliative care reasons.

### Statistical approach

Patient characteristics were compared between patients that did versus did not recover kidney function to dialysis independence within 1 year. An unadjusted Cox model was conducted for each predictor, with censoring for death.

Time dependent Cox regression analysis was performed to estimate the relationship of predictors with the primary outcome of recovery to dialysis independence within 1 year. To account for potential changes in dialysis prescription over the time period, UFR was used as a time-varying covariate. We generated a counting process data, in which each patient is represented by one row, and each row represents a 1 month interval during which the UFR could be changed while all other covariates remain constant.

## Results

### ESRD recovery trends

From 1996 through 2016 there were 2,166,429 incident dialysis patients, of whom 104,250 (4.8%) experienced kidney recovery. Over these 2 decades, the crude cumulative incidence of recovery increased gradually from 2.7% in 1996 to a peak of 6.3% in 2010, before decreasing the past several years (Table 1). Even with this recent trend, the cumulative incidence of recovery from the 5 most recent years represents a 91% increase in recovery compared to the first 5 years of the study period. The majority of recovering patients (62.5%) did so within the first 3 months after dialysis initiation.

**Table 1:** Trends in ESRD recovery, from 1996 to 2016

		Patients with Recovery by time from Dialysis Initiation					
Year	Incident ESRD Patients	0–3 months		4–12 months		Total within 1 year	
		N	%	N	%	N	%
1996	75,038	1138	1.52%	864	1.15%	2002	2.67%
1997	80,075	1323	1.65%	952	1.19%	2275	2.84%
1998	85,140	1381	1.62%	1016	1.19%	2397	2.82%
1999	88,735	1560	1.76%	1084	1.22%	2644	2.98%
2000	92,076	1749	1.90%	1294	1.41%	3043	3.30%
2001	95,763	2008	2.10%	1278	1.33%	3286	3.43%
2002	97,892	2277	2.33%	1538	1.57%	3815	3.90%
2003	100,145	2407	2.40%	1650	1.65%	4057	4.05%
2004	101,613	2661	2.62%	1762	1.73%	4423	4.35%
2005	103,751	2961	2.85%	1938	1.87%	4899	4.72%
2006	107,192	3167	2.95%	2096	1.96%	5263	4.91%
2007	107,176	3726	3.48%	2259	2.11%	5985	5.58%
2008	108,707	4099	3.77%	2249	2.07%	6348	5.84%
2009	112,300	4366	3.89%	2530	2.25%	6896	6.14%
2010	112,609	4426	3.93%	2700	2.40%	7126	6.33%
2011	110,420	4211	3.81%	2498	2.26%	6709	6.08%
2012	112,312	4496	4.00%	2505	2.23%	7001	6.23%
2013	115,070	4687	4.07%	2351	2.04%	7038	6.12%
2014	117,954	4413	3.74%	2154	1.83%	6567	5.57%
2015	121,471	4313	3.55%	2263	1.86%	6576	5.41%
2016	120,990	3746	3.10%	2154	1.78%	5900	4.88%
Total	2,166,429	65,115	3.01%	39,135	1.81%	104,250	4.81%

*ESRD* End-stage renal disease

### Patient characteristics

**Fig. 1:**
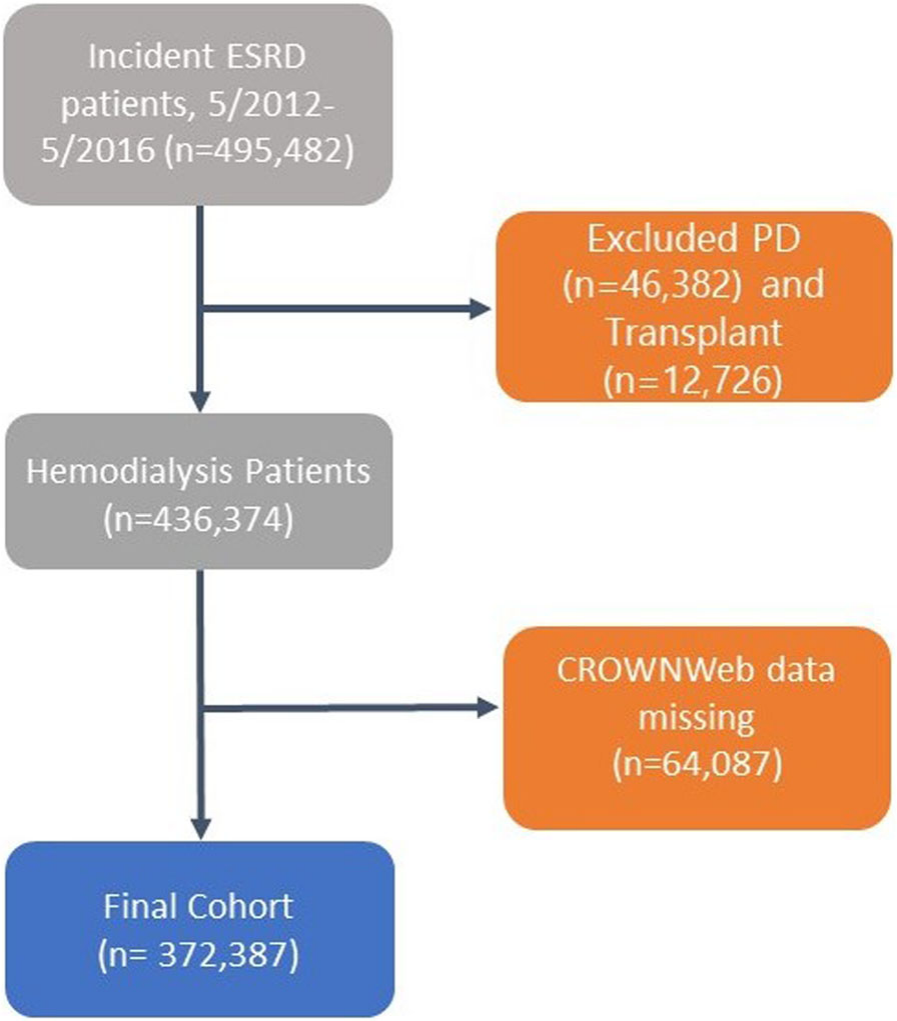
Derivation of analytic cohort

Between May 2012 and May 2016, we identified 372,387 incident HD patients for whom CROWNWeb data was available (Fig. 1). Among these, 16,930 (4.5%) recovered to dialysis independence within 1 year. Patient characteristics at time of ESRD incidence are shown in Table 2. A slightly higher proportion of men compared to women experienced recovery. White patients had the highest cumulative incidence of recovery at 5.4%, while 2.9% of Black patients recovered. Diagnoses of diabetes, congestive heart failure and cerebrovascular disease were each associated with lower likelihood of recovery. Evidence of prior nephrology care (such as prior use of erythropoiesis-stimulating agents or nephrology visits) was associated with lower likelihood of recovery.

**Table 2:** Patient characteristics by ESRD recovery status

	All Patients	Patients With Recovery	Patients Without Recovery	Univariate Cox model		
	372,387	16,930	355,457	Estimate	95% CI	*p*-value
Sex						
Male	214,906	9980 (4.6%)	204,926 (95.4%)	ref	ref	ref
Female	157,481	6950 (4.4%)	150,531 (95.6%)	0.95	0.92, 0.98	0.0013
Race						
White	247,388	13,306 (5.4%)	234,082 (94.6%)	ref	ref	ref
Black	102,499	3013 (2.9%)	99,486 (97.1%)	0.53	0.50, 0.58	< 0.0001
Native American	3541	98 (2.8%)	3443 (97.2%)	0.49	0.40, 0.60	< 0.0001
Asian	13,675	368 (2.7%)	13,307 (97.3%)	0.48	0.43, 0.53	< 0.0001
Pacific Islander	4413	100 (2.3%)	4313 (97.7%)	0.40	0.33, 0.48	< 0.0001
Other/unknown	871	45 (5.2%)	826 (94.8%)	0.96	0.71, 1.28	0.76
Mean age, years (SD)	372,387	63.0 (14.3)	63.4 (14.8)	1.001	1.00, 1.002	0.29
Age in years (category)						
0–44	40,796	1835 (4.5%)	38,961 (95.5%)	ref	ref	Ref
45–64	143,006	6524 (4.6%)	136,482 (95.4%)	1.03	0.98, 1.09	0.26
65+	188,585	8571 (4.5%)	180,014 (95.5%)	1.08	1.02, 1.13	0.004
Primary ESRD cause (category)						
Diabetes	179,438	5429 (3%)	174,009 (97%)	ref	ref	ref
Hypertension	112,091	3945 (3.5%)	108,146 (96.5%)	1.18	1.13, 1.22	< 0.0001
Glomerulonephritis	25,493	1381 (5.4%)	24,112 (94.6%)	1.78	1.68, 1.89	< 0.0001
Cystic Kidney	6286	70 (1.1%)	6216 (98.9%)	0.34	0.28, 0.45	< 0.0001
Other Urologic	4776	262 (5.5%)	4514 (94.5%)	1.82	1.64, 2.10	< 0.0001
Other Cause	34,961	4645 (13.3%)	30,316 (86.7%)	4.92	4.73, 5.11	< 0.0001
Unknown Cause	7868	661 (8.4%)	7207 (91.6%)	2.96	2.73, 3.20	< 0.0001
Missing Cause	1474	537 (36.4%)	937 (63.6%)	17.85	16.33, 19.50	< 0.0001
Comorbidities						
Diabetes Mellitus	219,004	8197 (3.7%)	210,807 (96.3%)	0.64	0.62, 0.67	< 0.0001
Atherosclerotic Heart Disease	58,333	2573 (4.4%)	55,760 (95.6%)	0.99	0.95, 1.03	0.60
Heart Failure	113,142	4560 (4.0%)	108,582 (96.0%)	0.87	0.84, 0.90	< 0.001
Hypertension	326,100	12,978 (4.0%)	313,122 (96.0%)	0.44	0.43, 0.46	< 0.001
Peripheral Vascular Disease	42,162	1947 (4.6%)	40,215 (95.4%)	1.04	0.99, 1.09	0.07
COPD	36,486	2038 (5.6%)	34,448 (94.4%)	1.32	1.26, 1.38	< 0.001
Cancer	26,453	1640 (6.2%)	24,813 (93.8%)	1.50	1.42, 1.58	< 0.001
Cerebrovascular Disease	32,996	1348 (4.1%)	31,648 (95.9%)	0.90	0.85, 0.95	0.0003
Pre-ESRD ESA Use						< 0.0001
No	205,728	10,216 (5%)	195,512 (95%)	ref	ref	ref
Yes	51,895	1103 (2.1%)	50,792 (97.9%)	0.42	0.39, 0.45	< 0.0001
Unknown	114,764	5611 (4.9%)	109,153 (95.1%)	0.99	0.96, 1.03	0.67
Pre-ESRD Nephrologist care						
No	95,181	7832 (8.2%)	87,349 (91.8%)	ref	ref	ref
Yes	223,610	5645 (2.5%)	217,965 (97.5%)	0.29	0.28, 0.30	< 0.0001
Unknown	53,596	3453 (6.4%)	50,143 (93.6%)	0.78	0.75, 0.82	< 0.0001
Insurance Coverage						
Medicare only	110,954	5096 (4.6%)	105,858 (95.4%)	ref	ref	ref
Employer only	41,771	1953 (4.7%)	39,818 (95.3%)	0.97	0.92, 1.02	0.27
Medicaid only	46,707	1854 (4%)	44,853 (96%)	0.83	0.78, 0.87	< 0.0001
Medicare and Medicaid	54,724	2087 (3.8%)	52,637 (96.2%)	0.82	0.78, 0.86	< 0.0001
Medicare and employer	22,187	994 (4.5%)	21,193 (95.5%)	0.97	0.90, 1.03	0.30
Medicare and other	46,250	2252 (4.9%)	43,998 (95.1%)	1.08	1.02, 0.13	0.004
None	30,019	1788 (6%)	28,231 (94%)	1.26	1.20, 1.33	< 0.0001
Other only	19,775	906 (4.6%)	18,869 (95.4%)	0.96	0.90, 1.03	0.30
Lab values at ESRD Initiation						
Serum Creatinine (mg/dL)	370,847	5.7 (3.8)	6.7 (10.3)	0.96	0.9, 0.91	< 0.0001
eGFR (mL/min/1.73m^2^)	367,099	11.7 (5.6)	10.1 (4.6)	1.07	1.07, 1.07	< 0.0001
Serum Albumin (g/dL)	251,335	3 (2.3)	3.2 (3.2)	0.73	0.71, 0.75	< 0.0001

*ESRD* End-stage renal disease, *ESA* Erythropoiesis-stimulating agent, *eGFR* Estimated glomerular filtration rate, *COPD* Chronic obstructive pulmonary disease

In terms of primary etiology for ESRD, the diagnostic categories with the highest cumulative incidence of recovery were “missing cause” (36.4%) and “other cause” (which includes acute tubular necrosis; 13.3%). When looking at specific diagnoses, the top 4 diagnoses by proportion of recovery were forms of acute kidney injury (AKI), each with > 20% of patients recovering (Table 3). Combined, these 4 diagnoses contributed to 16.0% of all recovery patients. Conversely, by absolute numbers the top ESRD diagnoses from which patients recovered were type 2 diabetes and hypertension, which together accounted for 51.9% of all recovery patients.

**Table 3:** Top diagnoses listed as primary cause of ESRD in patients with kidney function recovery, ranked by proportion and by absolute numbers

By Proportion Recovering			By Absolute Numbers Recovering		
Diagnosis	Total	Recovering	Diagnosis	Total	Recovering
Acute interstitial nephritis	783	215 (27.5%)	Diabetes Type 2	165,263	5046 (3.1%)
Postinfectious glomerulonephritis	256	64 (25.0%)	Hypertension, unspecified	108,470	3738 (3.4%)
Tubular necrosis (no recovery)	10,099	2359 (23.4%)	Tubular necrosis (no recovery)	10,099	2359 (23.4%)
Hemolytic uremic syndrome	313	68 (21.7%)	Missing or Uncertain	9053	1167 (12.9%)

### CROWNWeb data and treatment characteristics

Following dialysis initiation, albumin, calcium and hemoglobin were similar between patients that recovered versus did not recover (Table 4). Patients without recovery had higher phosphorus and parathyroid hormone levels. Patients without recovery also had higher monthly doses of erythropoiesis-stimulating agents and were more likely to have received intravenous iron supplementation. With regards to dialysis vascular access, a higher proportion of patients who had a catheter recovered than those with an arteriovenous fistula or graft (5.8% versus 1.0%, *p* < 0.001).

**Table 4:** Patient treatment characteristics and laboratory data as reported in CROWNWeb, by recovery status

Variable^a^	All		Patients With Recovery (*n* = 16,930)		Patients Without Recovery (*n* = 355,457)		*P*-value
	N	Mean (SD) or Proportion	N	Mean (SD) or Proportion	N	Mean (SD) or Proportion	
Albumin (g/dL)	353,182	3.5 (0.5)	16,012	3.4 (0.5)	337,170	3.5 (0.5)	< 0.001
Calcium (mg/dL)	355,196	8.8 (0.6)	16,226	8.9 (0.6)	338,970	8.8 (0.6)	< 0.001
Hemoglobin (g/dL)	357,569	10.4 (1.0)	16,365	10.3 (1.2)	341,204	10.4 (1.0)	< 0.001
ESA monthly dose (units)	299,143	43,949 (40196)	12,284	39,733 (40193)	286,859	44,130 (40187)	< 0.001
Ferritin	342,910	460 (328)	15,232	533 (373)	327,678	458 (325)	< 0.001
Iron % saturation	349,400	24.1 (8.8)	15,769	23 (10.1)	333,631	24.1 (8.8)	< 0.001
IV Iron administration							
Yes	212,999	59.7%	8534	52.7%	204,465	60.0%	< 0.001
No	144,016	40.3%	7647	47.3%	136,369	40.0%	
Phosphorus (mg/dL)	356,401	4.8 (1.2)	16,288	4.1 (1.0)	340,113	4.8 (1.2)	< 0.001
Parathyroid Hormone (pg/mL)	83,940	367 (314)	3755	221 (187)	80,185	374 (317)	< 0.001
Vascular Access Type							< 0.001
AVF/AVG	87,996	24.3%	915	5.5%	87,081	25.2%	
Catheter	255,931	70.8%	14,817	89.4%	241,114	69.9%	
Other	17,819	4.9%	837	5.1%	16,982	4.9%	
Treatment time per session (minutes)	335,737	223 (28)	15,996	219 (29)	319,741	223 (28)	< 0.001
Single pool Kt/V	353,328	1.5 (0.3)	16,101	1.5 (0.3)	337,227	1.5 (0.3)	< 0.001
Normalized protein catabolic rate (g/kg/d)	270,580	0.8 (0.2)	10,943	0.70 (0.2)	259,637	0.8 (0.2)	< 0.001
Pre-HD weight (kg)	335,856	84.3 (23.9)	16,004	85.8 (24.7)	319,852	84.2 (23.8)	< 0.001
Post-HD weight (kg)	335,855	82.3 (23.5)	16,004	84.2 (24.3)	319,851	82.2 (23.4)	< 0.001
Mean ultrafiltration rate (ml/kg/hr)	372,271	6.9 (3.1)	16,920	5.4 (3.5)	355,351	7.0 (3.1)	< 0.001
Quintile of ultrafiltration rate							< 0.001
1st (lowest) (<=4.3)	74,454	20.0%	6779	40.1%	67,675	19.0%	
2nd (> 4.3–5.9)	74,454	20.0%	3369	19.9%	71,085	20.0%	
3rd (> 5.9–7.3)	74,455	20.0%	2558	15.1%	71,897	20.2%	
4th (> 7.3–9.1)	74,454	20.0%	2117	12.5%	72,337	20.4%	
5th (> 9.1)	74,454	20.0%	2097	12.4%%	72,357	20.4%	

*ESA* Erythropoiesis-stimulating agent (includes all forms of ESA but predominantly epoeitin alfa during study period), *AVF* Arteriovenous fistula, *AVG* Arteriovenous graft, *HD* Hemodialysis. Note: collection of parathyroid hormone data in CROWNWeb ceased in April 2014

^a^Laboratory values are reported as the mean (SD) for continuous variables, and as a proportion for categorical variables. Data is aggregated for the first 3 months after dialysis initiation

**Fig. 2:**
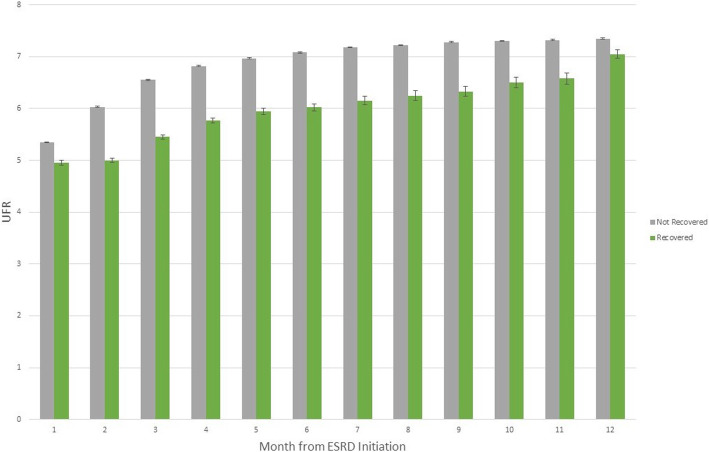
Mean ultrafiltration (UFR) rate by month, stratified by kidney function recovery

Dialysis treatment time per session was clinically similar between the recovery and non-recovery groups, as was single pool Kt/V. Pre- and post-dialysis weight were slightly higher in the recovery group compared to the non-recovery group. Mean UFR was significantly higher in the non-recovery group compared to the recovery group (7.0 vs 5.4 mL/kg/hr., *p* < 0.001). When categorized into quintiles, the proportion of patients recovering kidney function decreased with increasing quintile of UFR, ranging from 9.1 to 2.8% from lowest to highest quintile respectively (Table 4). When looking at monthly values, UFR was consistently lower in patients who eventually experienced recovery within 1 year compared to those that did not recover (Fig. 2).

In the adjusted model (Table 5), higher UFR over time remained significantly associated with lower likelihood of recovery. There was a stepwise reduction in hazard for recovery with each increasing quintile of UFR. Compared to the lowest quintile, patients with UFR in the top quintile had a 59% lower likelihood of recovery (HR 0.41, 95% CI 0.38–0.43). Higher eGFR was associated with recovery. Serum albumin was no longer a significant predictor. Calcium (HR 1.26, 95% CI 1.23–1.31), phosphorus (HR 0.57, 95% CI 0.56–0.58) and hemoglobin (HR 0.95, 95% CI 0.93–0.97) were all significantly associated with recovery.

**Table 5:** Multivariate time-dependent model for kidney recovery among incident hemodialysis patients

Variables	HR (95% CI)	*P*-value
Sex (ref = “male”)		
Female	0.96 (0.92,1.00)	0.050
Race (ref = “White”)		
Black	0.49 (0.47,0.52)	< 0.001
American Indian/Alaska Native	0.62 (0.48,0.80)	0.0003
Asian	0.64 (0.56,0.73)	< 0.001
Native Hawaii/Pacific Islander	0.57 (0.44,0.75)	< 0.001
Other	0.63 (0.39,1.01)	0.055
Age, years (ref = “0–44”)		
45–64	0.93 (0.86,1.00)	0.003
65+	0.74 (0.68,0.80)	< 0.001
Primary cause by category (ref = “Diabetes”)		
Cystic Kidney	0.46 (0.35,0.61)	< 0.001
Glomerulonephritis	1.82 (1.68,1.97)	< 0.001
Hypertension	1.12 (1.11,1.25)	< 0.001
Missing Cause	7.41 (4.03,13.62)	< 0.001
Other Cause	3.37 (3.17,3.57)	< 0.001
Other Urologic	1.52 (1.30,1.76)	< 0.001
Unknown Cause	2.02 (1.81,2.26)	< 0.001
Comorbidities		
Diabetes Mellitus	0.94 (0.90,0.99)	0.02
Atherosclerotic Heart Disease	0.97 (0.92,1.03)	0.28
Heart Failure	1.20 (1.14,1.25)	< 0.001
Hypertension	1.23 (1.17,1.29)	< 0.001
Peripheral Vascular Disease	0.89 (0.83,0.94)	< 0.001
COPD	0.83 (0.79,0.89)	< 0.001
Cancer	1.01 (0.95,1.08)	0.70
Cerebrovascular Disease	1.07 (1.00,1.15)	0.04
Institutionalized	0.95 (0.89,1.01)	0.10
Pre-ESRD ESA Use (ref = “no”)		
Yes	0.69 (0.63,0.74)	< 0.001
Unknown	1.06 (1.01,1.12)	0.03
Pre-ESRD Nephrologist care (ref = “no”)		
Yes	0.34 (0.32,0.35)	< 0.0001
Unknown	0.69 (0.64,0.73)	< 0.0001
Insurance Coverage (ref = “Medicare only”)		
Employer only	1.06 (0.99,1.15)	0.12
Medicaid only	0.89 (0.83,0.96)	0.003
Medicare and Medicaid	0.85 (0.80,0.91)	< 0.001
Medicare and employer	0.99 (0.91,1.08)	0.86
Medicare and other	1.01 (0.95,1.08)	0.68
None	0.95 (0.88,1.03)	0.25
Other only	0.94 (0.86,1.03)	0.20
Laboratory values		
Serum Creatinine (per mg/dL)	1.00 (0.99,1.01)	0.86
eGFR (mL/min/1.73m^2^)	1.05 (1.04,1.06)	< 0.001
Calcium (per mg/dL)	1.26 (1.22,1.31)	< 0.001
Albumin (per g/dL)	0.99 (0.95,1.04)	0.72
Phosphorus (per mg/dL)	0.57 (0.56,0.58)	< 0.001
Hemoglobin (per g/dL)	0.95 (0.93,0.97)	< 0.001
Quintile of ultrafiltration rate (ref = ‘1st/lowest’)		
2nd	0.70 (0.67,0.74)	< 0.001
3rd	0.58 (0.55,0.62)	< 0.001
4th	0.50 (0.47,0.53)	< 0.001
5th (highest)	0.41 (0.38,0.43)	< 0.001

*ESRD* End-stage renal disease, *ESA* Erythropoiesis-stimulating agent, *eGFR* Estimated glomerular filtration rate

## Discussion

Over the past two decades there has been a marked overall increase in kidney function recovery among incident ESRD patients. In this national analysis of USRDS data that included a first examination of CROWNWeb data in this context, we identified clinical characteristics and potentially modifiable treatment factors associated with recovery to dialysis independence, a highly relevant, patient-centered outcome. Our findings can help clinicians appropriately risk stratify incident ESRD patients.

We identified several factors associated with kidney recovery, including demographic factors such as female sex and younger age. Most notably, the primary ESRD diagnoses associated with the greatest likelihood of kidney recovery were acute tubular necrosis, acute interstitial nephritis, hemolytic uremic syndrome, and post-infectious glomerulonephritis – each with 20% or higher recovery. Although non-modifiable, these characteristics can help clinicians risk stratify for the purposes of both monitoring and potentially changing management. For patients most likely to recover, clinicians may choose to defer declaration of ESRD in favor of continued observation for recovery, which has been made easier by the implementation of the Trade Bill allowing AKI related maintenance dialysis at ESRD facilities [7]. In these cases, clinicians may apply recently proposed protocols emphasizing closer monitoring of residual kidney function and increased attention to intradialytic hemodynamic stability [8]. Patients with a favorable recovery profile should be properly educated about signs of kidney recovery and avoidance of potential nephrotoxic exposures. Risk stratification is also important to identify those least likely to recover. Examples include earlier placement of an arteriovenous fistula for vascular access, or earlier referral for transplant evaluation. Thus, accurate risk assessment allows both the opportunity to optimize likelihood of recovery and also the efficient use of resources for those unlikely to recover.

While AKI-related primary diagnoses had the highest cumulative incidence of recovery, they accounted for only about 20% of all recovering ESRD patients. Conversely, patients with ESRD attributed to type 2 diabetes and hypertension comprised slightly more than 50% of all ESRD patients who recovered. Presumably, these patients had an AKI component on top of other comorbidities that was either unrecognized or underestimated. Importantly, when completing the CMS 2728 ESRD certification form, clinicians are only able to pick one primary diagnosis as the cause of ESRD. Further, there is inherent subjectivity in choosing the primary diagnosis, which can predispose to inaccuracies [9]. If an underlying AKI is a component of those who recover, this might not be reported and thus overlooked by clinicians. This oversight might further be accentuated in the transition from hospital to ESRD facilities providing dialysis treatment. Clinically, there is a need to better identify this AKI component in order to recognize the potential for recovery. One possible systems-based solution could be to add a “secondary diagnosis” field to the ESRD certification form, where an acute component could be identified. This change might allow clinicians to identify and treat those with an underlying AKI with a more comprehensive approach. The use of a secondary diagnosis might also improve the handoff between hospital and ESRD facilities.

Another area that warrants further inquiry is the apparent racial inequality in recovery. After adjusting for other characteristics, all race groups had a decreased recovery of kidney function when compared to white patients; this difference was most pronounced with Black patients, who had a 51% lower likelihood of recovery. There are a few possible explanations for this finding. Biologically, the high risk alleles of the APOL1 gene have been implicated in the excess risk for ESRD observed in Black compared to white patients [10]. Although a link between APOL1 and AKI has not been established, [11] it is conceivable that a severe AKI episode could accelerate an underlying ESRD predisposition in high risk patients. Beyond genetic explanation, it is important to discuss the impact of health inequality, and perhaps even unconscious bias, affecting this population. Knowing that Black patients are generally more likely to have ESRD, providers may be predisposed to overlooking the possibility of recovery and/or have a lower threshold for designating a patient as ESRD in the first place. Patient recovery may also be affected by socioeconomic disparities manifested by lower health literacy and education levels. Social determinants of health disproportionately affect Black patients, and have been shown to be prevalent factors in those with kidney disease [12]. Previous studies have also demonstrated a link between lower health literacy and adverse outcomes in patients with CKD and ESRD [13]. Further evaluation is needed to explore the role of social determinants of health in kidney recovery, as these may represent an important opportunity to help reduce the racial disparities observed in our study.

In this study, which to the best of our knowledge is the first exploration of dialysis treatment data available in the USRDS and recovery, we found that higher quintiles of UFR were associated with a progressively lower likelihood of kidney recovery. Being an observational study, we cannot derive a causal relationship between UFR and kidney recovery, especially since CROWNWeb does not capture potentially important confounders such as residual kidney function and urine output. Nonetheless, higher UFR is a well-established risk factor for intradialytic hemodynamic instability [14] which can contribute to organ perfusion damage, providing a plausible mechanism for hindering kidney recovery. Indeed, several studies have reported an association between higher UFR and risk for adverse outcomes such as cardiovascular and all-cause mortality in hemodialysis patients [15, 16]. A recent study linked higher UFR with both higher mortality and greater loss of residual kidney function; notably, this risk was increased even at UFR values of 6–10 mL/kg/hr. when compared to < 6 mL/kr/hr [17]. Lower UFR values (< 10 mL/kg/hr) have traditionally been considered somewhat “safe”, which emphasizes the importance of monitoring hemodynamic variables. Indeed, a single-center study of patients with AKI requiring outpatient dialysis similarly noted that more frequent episodes of intradialytic hypotension were associated with lower likelihood of recovery to dialysis independence [18]. Our findings build upon this literature by identifying higher UFR as an important and potentially modifiable risk factor for kidney function non-recovery among incident ESRD patients.

Due to the increased recognition of the harms of intradialytic hypotension and link with higher UFR, the Centers for Medicare and Medicaid Services (CMS) adopted UFR as a reporting quality measure beginning in 2020 [19]. Our findings further highlight the importance of this component of dialysis prescription and would seem to support the recommendations from the Acute Dialysis Quality Initiative’s recently published “WATCH-ME” protocol: Weight assessment, Access, Teaching (patient education), Clearance (assessment of residual kidney function), Hypotension avoidance, and Medication review [8]. This protocol emphasizes the importance of weight management and avoidance of hypotension through permissive hypervolemia if necessary. Of note, excessive hypervolemia has also been associated with worse outcomes, [20] so a balance of these extremes is key in weight management for these patients. Whether application of these principles to incident ESRD patients who are more likely to recover kidney function may result in increased recovery has not been evaluated. However, observational studies have noted that facilities with protocols specifically aimed at reducing intradialytic hemodynamic instability are associated with improved patient outcomes compared to facilities without such protocols [21]. In particular, patients with a primary ESRD diagnosis of AKI require close observation for evidence of renal recovery, and more attention to weight changes and hypotension might provide a good starting point.

There are several limitations to our study. Most notably, the observational design and lack of data on residual kidney function and urine volume limit any causal inferences between the observed higher UF rates and lower likelihood of renal recovery. Nonetheless, our findings are plausible and in line with expert guidelines regarding optimal approaches to dialysis prescription in patients with AKI. We encourage dialysis organizations which have access to more granular treatment data to further explore and confirm our study’s findings. Another limitation is the inherent subjectivity in determining ESRD status and assigning a primary cause of this diagnosis. It is not feasible to do a nephrologist-level analysis, but it is conceivable that individual practice patterns could significantly influence recovery rates. Our study period was prior to recent regulatory changes (i.e. passage of the Trade Bill as discussed above) which have potentially significantly changed the approach to ESRD diagnosis in patients with AKI; future analyses are needed to assess the impact of these changes. Lastly, our primary outcome of kidney function recovery may have incomplete ascertainment due to lack of recognition. We hope that the results of this study will increase awareness of the potential for recovery and reduce any missed opportunities.

## Conclusions

Nearly 1 in 20 incident ESRD patients will recover to dialysis independence within 1 year. In this study we identified several predictors of kidney recovery, including a potential link between higher ultrafiltration rate and reduced likelihood of recovery. Clinicians can use this information to risk stratify patients and consider delaying ESRD designation and/or closer monitoring for recovery, particularly among patients with a primary ESRD diagnosis in an AKI category.

## Data Availability

The datasets analyzed during the current study are available from the United States Renal Data System (USRDS), which is a publicly available data source. Requests for datasets can be made at https://usrds.org/for-researchers/.

## Abbreviations

AKI: Acute kidney injury
CKD: Chronic kidney disease
CMS: Centers for Medicare & Medicaid Services
CROWNWeb: Consolidated Renal Operations in a Web Enabled Network
ESRD: End-stage renal disease
HD: Hemodialysis
UFR: Ultrafiltration rate
UNOS: United Network for Organ Sharing
USRDS: United States Renal Data System
